# Clinical Applications of MicroRNAs in Acute Myeloid Leukemia: A Mini-Review

**DOI:** 10.3389/fonc.2021.679022

**Published:** 2021-08-11

**Authors:** Bhavana Bhatnagar, Ramiro Garzon

**Affiliations:** ^1^Division of Hematology and Medical Oncology, West Virginia University Cancer Institute, Schiffler Cancer Center, Wheeling, WV, United States; ^2^Division of Hematology, Department of Internal Medicine, The Ohio State University Comprehensive Cancer Center, Columbus, OH, United States; ^3^The Ohio State University Comprehensive Cancer Center, Columbus, OH, United States

**Keywords:** acute myeloid leukemia, AML, miR, microRNA, review

## Abstract

MicroRNAs (miRs) are short non-coding RNAs, typically 18-25 nucleotides in length, that are critically important, through their direct effects on target mRNAs, in a variety of cellular processes including cell differentiation, proliferation and survival. Dysregulated miR expression has been identified in numerous cancer types including acute myeloid leukemia (AML). From a clinical standpoint, several miRs have been shown to associate with prognosis in AML patients. Furthermore, they also carry the potential to be used as biomarkers and to inform medical decision making. In addition, several preclinical studies have provided strong rationale to develop novel therapeutic strategies to target miRs in AML. This review will focus on potential clinical applications of miRs in adult AML and will discuss unique miR signatures in specific AML subtypes, their role in prognostication and response to therapy, as well as miRs that are promising therapeutic targets and ongoing clinical trials directed towards targeting clinically relevant miRs in AML that could allow for improvements in current treatment strategies.

## Introduction

Acute myeloid leukemia (AML) is a clinically and biologically heterogeneous disease characterized by a number of recurring, sequential genetic alterations that result in a block in differentiation and expansion of immature myeloid precursors. In addition to the cytogenetic and molecular changes that are common in AML (1, 2), dysregulated microRNAs (miRs) have also been identified to play a critical role in leukemogenesis (3–5).

MicroRNAs are small non-coding RNAs, typically 18-25 nucleotides in length that affect the post-transcriptional function of specific mRNAs and their resultant protein targets (6, 7). Previous work has revealed that dysregulated expression of even of a single miR targets multiple mRNAs and modulates the function of numerous cellular pathways (8). Clinically, miR expression patterns have the potential to inform medical decision making. For example, miR expression can be used to differentiate between acute leukemias of ambiguous lineage (9, 10), refine current AML prognostic classification systems (5, 11), potentially detect progression of myelodysplastic syndrome (MDS) to AML (12) and to detect measurable residual disease (MRD) (13, 14).

Over the past decade, several miRs have been shown to be aberrantly under or overexpressed in AML. Although miR expression signatures have been shown to be distinct in specific subtypes of AML and to correlate with prognosis (5, 11, 15–18), miR expression profiling is not yet incorporated into routine clinical practice (19). Additionally, several pre-clinical studies have provided proof-of-concept that miRs are actionable therapeutic targets. However, while miR-directed therapies have proven to be successful in other disease types, most notably in hepatocellular carcinoma (20), cutaneous T-cell lymphoma (21) and diffuse large B-cell lymphoma (22), these therapies remain in an early stage of translation in AML. This review will summarize key clinical applications of microRNAs as they pertain to the management of patients with AML (**Table 1**) and will also discuss the current status of miR-directed therapies and barriers to implementation.

**Table 1:** Key microRNAs and their roles in AML.

MicroRNAs with context-dependent roles		
miR	Significance	Reference no.
**miR-9a**	Overexpression in *KMT2A*-rearranged AML associates with oncogenic role	(23)
	Overexpression in pediatric AML with t(8;21) associates with tumor suppressor role	(24)
**miR-125b**	Level of expression (high *vs* low) promotes either lymphoid or myeloid malignancies	(25)
**miR-126**		(26)
**miR-155**	High level of over expression (>10-fold) associates with tumor suppressor role. Low or intermediate levels of overexpression associate with tumor promotor role.	(27)
**MicroRNA expression patterns in specific AML subtypes**		
**miR(s)**	**Significance**	**Reference no.**
**miR-10a, miR-10b, miR-196a and miR-196b (HOX cluster)**	Upregulated in *NPM1*-mutated AML	(28)
**miR-155, miR-144 and miR-451**	Overexpression of miR-155 and lower expression of miR-144 and miR-451 observed in CN-AML with FLT3-ITD.	(29)
**MicroRNAs that associate with response to chemotherapy or transplant**		
**miR(s)**	**Significance**	**Reference no.**
**miR-29b**	High pre-treatment expression levels associate with improved responses to decitabine	(30)
**miR-29c**	Low expression levels predict for improved response to azacitidine and high levels associate with relapse.	(31)
**miR-126**	Low expression associates with worse response rates to azacitidine.	(32)
**miR-99a**	High expression associates with worse survival post-transplant	(33)
**miR-425**	High expression associates with improved response to consolidation chemotherapy and low expression associates with better response to transplant.	(34)
**MicroRNAs with prognostic relevance**		
**miR**	**Significance**	**Reference no.**
**miR-155**	High expression in CN-AML associates with lower complete remission rates and overall survival	(35)
**miR-181**	High expression in CN-AML associates with higher remission rates and overall survival, especially in patients with NPM1 mutations and *FLT3*-ITD.	(36, 37)
**miR-25**	Higher levels associated with improved survival	(37)
**miR-362**	Higher levels associated with worse overall survival	(37)
**MicroRNAs that have been studied as therapeutic targets**		
**miR**	**Significance**	**Reference no.**
**miR-126**	Targetable with nanoparticle compound antagomiR-126	(38–40)
**miR-29b**	Transferrin-conjugated nanoparticle drug delivery system shown to increase miR-29b levels to enhance responses to treatment	(41)
**miR-155**	Targetable with NEDD8 inhibitors and Silvestrol	(42)
**miR-150**	Targetable with *FLT3* guided-mmiR-150 nanoparticles	(43)

CN-AML, cytogenetically normal acute myeloid leukemia.

## MicroRNAs and Their Key Functions in Acute Myeloid Leukemia

MicroRNAs modulate a large variety of cellular pathways that are critical for leukemogenesis such as cell differentiation, proliferation, epigenetic regulation, and stem cell function and survival (6). MiRs exert their effects at the post-transcriptional level by binding to the complementary 3’ untranslated region (3’ UTR) of target mRNAs and marking them for cleavage or destruction thereby inhibiting translation (6, 7, 44, 45). Under normal physiological conditions, miRs are essential for maintaining hematopoiesis (46) including stem cell function and lineage commitment (47, 48). Thus, it is not surprising that miR dysregulation plays a critical role in the initiation and maintenance of leukemogenesis.

Alterations of miR expression in AML result directly from genomic deletions, translocations, amplifications, and/or from epigenetic alterations through aberrant transcription factors, or oncogenic fusion proteins and global/specific chromatin accessibility changes. Dysregulated miRs can either function as oncogenes (oncomiRs) or as tumor suppressors. Interestingly, it is not uncommon for a specific miR to play opposing roles depending on the cellular context and type of leukemia. In *KMT2A*-rearranged AML, for instance, miR-9a overexpression has been reported to act as an oncogene (23), but, in pediatric patients with t(8;21), overexpression of miR-9a plays a tumor suppressor role (24).

In addition to this context-dependent duality in function, several studies have demonstrated that the magnitude of expression levels of the same miR (high *vs* low) can lead to different outcomes when miR expression levels are artificially controlled. For instance, miR-125b promotes either lymphoid or myeloid malignancies depending on its level of expression (25) and dysregulated miR-126 has been shown to promote AML in mouse models due to both over expression and loss of function in concert with t (8, 21) fusion genes (26). Additionally, miR-155 has been shown to function as a tumor suppressor when there is a high level of over expression (>10-fold) and as a tumor promoter when overexpressed to low (< 5 fold) or intermediate levels (5-10 fold) (27). Thus, cellular context is an important consideration when considering the biological functions of miRs in AML.

## MicroRNA Expression Patterns Define Specific Subtypes of AML and Associate With Outcome

MicroRNA profiling has been assessed in several AML subtypes and characteristic miR signatures have been observed in specific AML cytogenetic and molecular subgroups (3, 4, 11, 16, 49). One of the first studies to perform comprehensive miR profiling, using a micro-array based assay, on 122 AML patient samples from specific cytogenetic and molecular subgroups identified distinct miR expression profiles associated with cytogenetics and recurrent molecular alterations. Furthermore, this same study demonstrated that overexpression of specific miRs (miR-191 and miR-199a) correlated with prognosis (11). Since then, several groups reported miR expression signatures in several other cytogenetic or molecular subgroups. Older (>60 years) and younger AML patients with *NPM1* mutations, displayed upregulation of HOX genes and their associated miRs embedded within the HOX cluster, including miR-10a, miR-10b, miR-196a and miR-196b (28). Cytogenetically normal AML (CN-AML) patients with *FLT3*-ITD, were also found to have a distinct miR signature, which included overexpression of miR-155 and lower expression of miR-144 and miR-451 (29). Another study profiled miRs in 215 newly diagnosed AML cases using reverse-transcription-polymerase chain reaction (RT-PCR-based) and identified unique miR expression patterns in AML patients with t(8;21), t(15;17), inv(16), *NPM1*, and *CEBPA* mutations (3). This, subsequently allowed for identification of specific miRs that were differentially expressed within these subtypes, suggesting that miR expression could potentially be used to classify and characterize AML on a deeper and more comprehensive level than with cytogenetic or molecular data alone (3). In a more recent study, approximately 1000 miRs were sequenced from AML samples and compared to peripheral blood samples collected from control subjects which revealed a higher number of aberrantly expressed miRs in *NPM1*-mutated and *FLT3*-mutated AML patients compared to control subjects (50). Several of these miRs had not been previously described in association with these leukemia subtypes (50).

It is worth noting that, although the roles of numerous miRs have been evaluated in AML, only a few have been validated across multiple studies, such as the upregulation of miR-10a, miR-10b and miR-155 in *NPM1* mutated and *FLT3*-ITD AML, respectively.

## MicroRNAs Can Help Predict Response to Chemotherapy

MicroRNA expression has also been used to determine the effects of response to hypomethylating agents (HMAs) in AML patients (32, 51). Blum and colleagues, for instance, were able to demonstrate, in a pivotal phase 2 study, that older AML patients with higher pre-treatment levels of miR-29b, which is known to target DNA methyltransferases, were more likely to achieve a clinical response following induction chemotherapy with 10 days of the HMA, decitabine (30). A subsequent preclinical study using the histone deacetylase (HDAC) inhibitor, AR-42, in combination with decitabine revealed that AR-42 priming was able to increase miR-29b expression levels and enhance the antileukemic activity of decitabine in AML cell lines (52). However, the phase I clinical study that followed was unable to show improved responses using this approach (53).

Azacitidine, another important HMA employed for the treatment of AML in older and/or unfit adults has also been evaluated within the context of miR profiling. Another member of the miR-29 family, miR-29c, has been reported to be predictive of favorable responses to azacitidine at low expression levels, whereas upregulated miR-29c, was associated with higher rates of relapse (31). Several other groups have demonstrated that azacitidine responders have differing miR expression patterns compared to azacitidine non-responders (32, 51). Solly and colleagues compared differences in expression between 754 miRs in azacitidine-resistant and azacitidine-sensitive cell lines and were able to show that low expression levels of miRs that affected the function of the DNA methyltransferase, DNMT1, could potentially account for azacitidine resistance in AML patients. Low expression levels of miR-126, an anti-DNMT1 miR, were found to have the most adverse impact on response rates (32).

In addition, certain miRs have associated with clinical responses following allogeneic hematopoietic stem cell transplant (alloHSCT). For example, overexpression of miR-99a, which correlates with inferior prognosis in AML patients (17) was also studied in 74 AML patients who received alloHSCT. In this setting, high miR-99a expression associated with worse event-free survival (EFS) and overall survival (OS). Furthermore, it was identified as an independent risk factor for inferior EFS and OS in AML patients who received transplant, suggesting that miR-99a expression could be used to predict for unfavorable outcome (33). In another study, miR-425 expression levels and impact on EFS and OS were studied in 162 AML patients who received either consolidative chemotherapy or transplant (34). In this report, AML patients younger than age 60 years with high miR-425 expression levels had improved EFS (*P*=0.001) and OS (*P*=0.001) compared to low miR-425 expressers whereas low expressers had improved responses to alloHSCT (P<0.001), thereby supporting the role of miR-425 expression levels in order to select the most effective consolidation therapy in younger patients.

## MicroRNAs Carry Prognostic Relevance in AML

Many studies have demonstrated that under or overexpression of specific miRs correlates with prognosis in AML patients, particularly in patients with CN-AML. In a seminal study by Marcucci and colleagues, microRNA expression profiling in younger (below age 60 years) CN-AML patients identified 12 miRs, five of which were in the miR-181 family, from which a weighted miR summary value could be derived that inversely associated with EFS (5). In a separate study of 363 patients with CN-AML, high expression of miR-155 correlated with inferior outcomes compared to patients with low miR-155 expression (35). This study was also the first to demonstrate that miR-155 overexpression was an independent prognostic predictor of lower complete remission (CR) rate and shorter OS (35). Similarly, miR-181a expression was also studied in a cohort of younger CN-AML patients and was found to associate with improved CR rates, OS and disease-free survival (DFS). Notably, patients with *FLT3*-ITD or wild-type *NPM1* with high miR-181a expression experienced higher CR rates and improved DFS and OS (36). Since these studies were published, numerous other miRs have been studied and their prognostic impact has been well described in other reviews (4, 45, 54). In a more recent study, TCGA data was analyzed to identify miRs with the greatest prognostic value in 179 non-M3 AML patients. Out of 705 miRs that were studied, miR-181a-2, miR-25 and miR-362 expression levels correlated the most with prognosis (37).

## MicroRNAs as Therapeutic Targets in AML

The field of miR therapeutics was originally pioneered in patients with chronic hepatitis C virus (HCV) infection. Miravirsen, an anti-miR-122 locked nucleic acid (LNA) naked oligonucleotide was evaluated in a landmark phase 2 study of 36 patients with chronic HCV and demonstrated a dose-dependent reduction in viral RNA titers and no evidence of viral resistance (20). These findings paved the way for miR-directed therapies in cancer. For example, MRG-106, the LNA miR antagonist to miR-155, was the first of such therapies to be successfully employed in patients with cutaneous T-cell lymphoma (21).

In addition, several miRs serve as potential targets for novel therapeutic approaches in a broad array of diseases. At present, a review of the ClinicalTrials.gov site shows that over 850 studies have been registered that incorporate miRs as biomarkers or as therapeutic targets. **Table 2** provides investigational clinical studies that utilize specific therapeutics to target specific miRs.

**Table 2:** MicroRNA-directed clinical trials.

Study Title (NCT)	miR under investigation	Disease(s)	Status
A Multicenter Phase I Study of MRX34, MicroRNA miR-RX34 Liposomal Injection (NCT01829971)	miR-34	Primary Liver Cancer, SCLC, Lymphoma, Melanoma, Multiple Myeloma, Renal Cell Carcinoma, NSCLC	Terminated (5 immune mediated adverse events)
A Phase 1, Open-Label Study to Evaluate the Safety, Pharmacodynamics, and Pharmacokinetics of RG-012 for Injection, Including Its Effect on Renal microRNA-21, in Subjects With Alport Syndrome (NCT03373786)	miR-21	Alport Syndrome	Completed
MesomiR 1: A Phase I Study of Intravenously Administered Epidermal Growth Factor Receptor -Targeted, EnGeneIC Delivery Vehicle (EDV)-Packaged, miR-16 Mimic (TargomiRs) for Patients With Malignant Pleural Mesothelioma (MPM) and Advanced Non-Small Cell Lung Cancer (NSCLC) Failing on Std Therapy (NCT02369198)	miR-16	Mesothelioma and NSCLC	Completed
A Phase 1, Randomized, Double-blind, Placebo-controlled, Single and Multiple Ascending Dose-escalation Study to Investigate the Safety, Tolerability, Pharmacokinetics, and Pharmacodynamic Activity of MRG-110 Following Local Intradermal Injection After Skin Excisional Wound Creation in Normal Healthy Volunteers (NCT03603431)	miR-92a	Normal healthy volunteer	Completed
A Phase 2, Double-blind, Placebo-Controlled Study to Investigate the Efficacy, Safety and Tolerability of MRG-201 Following Intradermal Injection in Subjects With a History of Keloids (NCT03601052)	miR-92	Keloids	Active, not recruiting
A Phase I/II, Randomized, Double-blind, Sham Control Study to Explore Safety, Tolerability, and Efficacy Signals of Multiple Ascending Doses of Striatally-Administered rAAV5-miHTT Total Huntingtin Gene (HTT) Lowering Therapy (AMT-130) in Early Manifest Huntington Disease (NCT04120493)	miHTT	Huntington’s Disease	Recruiting
A Placebo-controlled, Double-blind, Randomized, Single Dose, Dose Escalating Trial in Healthy Men to Evaluate the Safety, Tolerability, Pharmacokinetics and Pharmacodynamics of SPC3649 (Miravirsen) (NCT00688012)	miR-122	Healthy volunteers	Completed
SOLAR: A Phase 2, Randomized, Open-label, Parallel-group, Active Comparator, Multi-center Study to Investigate the Efficacy and Safety of Cobomarsen (MRG-106) in Subjects With Cutaneous T-Cell Lymphoma (CTCL), Mycosis Fungoides (MF) Subtype (NCT03713320)	miR-155	CTCL/Mycosis Fungoides	Active, not recruiting
A Multicenter Phase 1B Pharmacodynamics Study of MRX34, MicroRNA miR-Rx34 Liposomal Injection, in Patients With Advanced Melanoma and Biopsy Accessible Lesions (NCT02862145)	miR-34	Melanoma	Withdrawn

There are mainly two strategies to silence an oncogenic overexpressed miR (oncomiR): 1) utilization of antisense oligonucleotides (ASOs) and 2) indirect targeting of the oncomiR by using small molecules or other agents that target the transcription or processing of the miR itself. With respect to the first approach, ASOs are oligonucleotides that are complementary to the target sequence and degrade or block the transcript by base-pairing. The initial hurdles in ASO development include the short life, and degradation of ASOs. To overcome this problem chemical modifications such as the addition of 2’-O-methyl groups and LNAs have greatly improved the stability of ASOs as well as their binding affinity and nuclease resistance. Another important issue is addressing optimal delivery of the ASOs to the target cells, more specifically, the AML blasts that are in blood and bone marrow. Over the past several years there has been a push to develop nanoparticle-based drug delivery systems for ASOs, with some success, including the relatively recent Food and Drug Administration approval of the RNA-inhibitor (RNAi) based therapy patisiran in 2018.

The second approach to target an oncomiR is to use small molecules or other agents that affect the transcription or stability of the miR. These indirect strategies are nonspecific since they act on transcription factors that regulate the miR or the epigenetic machinery. For example, one approach that our own group evaluated in a phase 1 study, based on promising pre-clinical data (55) was to use bortezomib and sorafenib priming prior to decitabine therapy in order to increase miR-29b expression in AML blasts to and subsequently improve disease responses (ClinicalTrials.gov Identifier: NCT01861314).

The strategies to restore the expression of a tumor suppressor miR include also an indirect approach using small molecules and other agents and a direct strategy using miR mimics delivered by adenovirus or nanoparticles. In hepatocellular carcinoma, peptide-based nanoparticles have been manufactured to deliver miR-199a-3p, a tumor suppressor, successfully in animal models (56).

Given their pleotropic nature, miR directed therapies offer an attractive treatment approach in AML as well. Several pre-clinical studies have identified candidate miRs that may be amenable to future targeted therapies. Dorrance and colleagues, for instance, were able to confirm that high miR-126 expression levels correlated with adverse outcomes in older CN-AML patients (38). Additionally, miR-126 overexpression was found to associate with a leukemia stem cell (LSC) gene expression profile, suggesting that miR-126 directed therapies contain potential to eradicate LSCs and improve disease responses (38–40). *In vivo* studies from the same group demonstrated the feasibility of direct targeting of miR-126 using the novel nanoparticle compound antagomiR-126 (38).

Similarly, Huang and colleagues, were able to demonstrate that a novel transferrin-conjugated nanoparticle drug delivery system could be effectively utilized to increase miR-29b levels and that priming AML cells with this agent enhanced responses to decitabine (41).

Preclinical studies evaluating the activity of NEDD8 activating enzyme (NAE) inhibition on miR-155 expression in AML cell lines demonstrated that miR-155 could be downregulated through disruption of binding of NF-KB to the miR-155 promoter, suggesting that NEDD8 inhibitors, such as pevonedistat, may be novel treatment options for AML associated with high miR-155 expression (42). Another preclinical study evaluated the activity of a natural compound, silvestrol, in *FLT3*-ITD and *FLT3*-wild type AML and demonstrated potent antileukemic activity and marked downregulation of miR-155, which is typically concurrently regulated in patients with AML and *FLT3*-ITD (43).

Another miR that is potentially targetable is miR-150, a tumor suppressor and negative regulator of *FLT3*. MiR-150 has also been described as a promoter of myeloid differentiation, therefore, low or absent expression of miR-150 leads to maturation arrest in AML cells (57). *FLT3* guided-mmiR-150 nanoparticles, in preclinical studies, were able to penetrate the bone marrow and suppress the growth of *FLT3*-mutated AML cells (58).

Taken together, these pre-clinical studies provide important proof-of-concept that miRs can be targeted and their function can be altered with miR-directed therapies. However, translation of these findings to AML patients has been met with significant challenges.

## Discussion

Essentially, over the past 15 years, the discovery of miRs and their numerous functions under both normal and pathogenic conditions have provided important biologic insights pertaining to their roles in the development of both malignant and non-malignant disease states.

In AML, specifically, several elegant studies have provided strong rationale to utilize miR expression profiling, in addition to current genetic and molecular testing, in order to better characterize an individual’s specific leukemia. Other groups have also aptly demonstrated that specific miRs can help predict responses to commonly used AML-directed chemotherapy regimens or allogeneic stem cell transplant (30–34, 51). Furthermore, Shivarov and colleagues have suggested that certain overexpressed miRs (miR-19a, miR-181a, miR-17, miR-181b, miR-221, miR-326, and miR-222) can potentially be used for PCR-based MRD detection following intensive induction chemotherapy, though larger studies are needed to validate these findings (14). Haferlach and colleagues led an in international effort, through the Microarray Innovations in Leukemia study, and were able to demonstrate the feasibility of performing whole genome expression profiling in over 3,000 patients with acute leukemia and MDS and also reported a median sensitivity exceeding 99% in classifying at least 14 subtypes of leukemia (26).

Despite these results as well as the unequivocally impactful role of miR expression profiling in the management of AML patients, this platform is not yet routinely incorporated into clinical practice, nor has it been included in commonly used AML risk stratification systems (2, 59). Many of the barriers precluding widespread use and integration are largely related to lack of standardized approaches regarding the optimal sample type as well as the variability in platforms used for miR profiling (array based, RT-PCR or NGS) and the sensitivity of each of these techniques (13, 60). Novel strategies such as direct measurement of the miR molecules like nanoString may circumvent this problem. As miRs continue to be explored and validated in AML, it is likely that, over time, the most optimal way to integrate them into clinical management will become better defined and that miR expression profiling will, at some point, become a more standard aspect of disease classification.

Unfortunately, although several miRs are promising pharmacologic targets in AML, miR-directed treatments have not yet been studied in clinical trials yet for AML patients. There are still several barriers to drug development in this area including concerns for off target effects, toxicity and target delivery to blasts. Hopefully, with proper design of ASOs or mimics and with better delivery vehicles there will soon be phase 1 trials in AML targeting miRs.

## Author Contributions

BB and RG wrote, revised, and edited this manuscript. All authors contributed to the article and approved the submitted version.

## Conflict of Interest

The authors declare that the research was conducted in the absence of any commercial or financial relationships that could be construed as a potential conflict of interest.

## Publisher’s Note

All claims expressed in this article are solely those of the authors and do not necessarily represent those of their affiliated organizations, or those of the publisher, the editors and the reviewers. Any product that may be evaluated in this article, or claim that may be made by its manufacturer, is not guaranteed or endorsed by the publisher.
